# Tracking Senior Fall and Fall-Related Injury EMS Calls to Target Fall Prevention Programs, Salt Lake County, Utah

**DOI:** 10.5888/pcd16.180462

**Published:** 2019-04-18

**Authors:** Jiyoung Byun, Jenny Robertson

**Affiliations:** 1 Epidemiology Bureau, Salt Lake County Health Department, Salt Lake City, Utah

**Figure:**
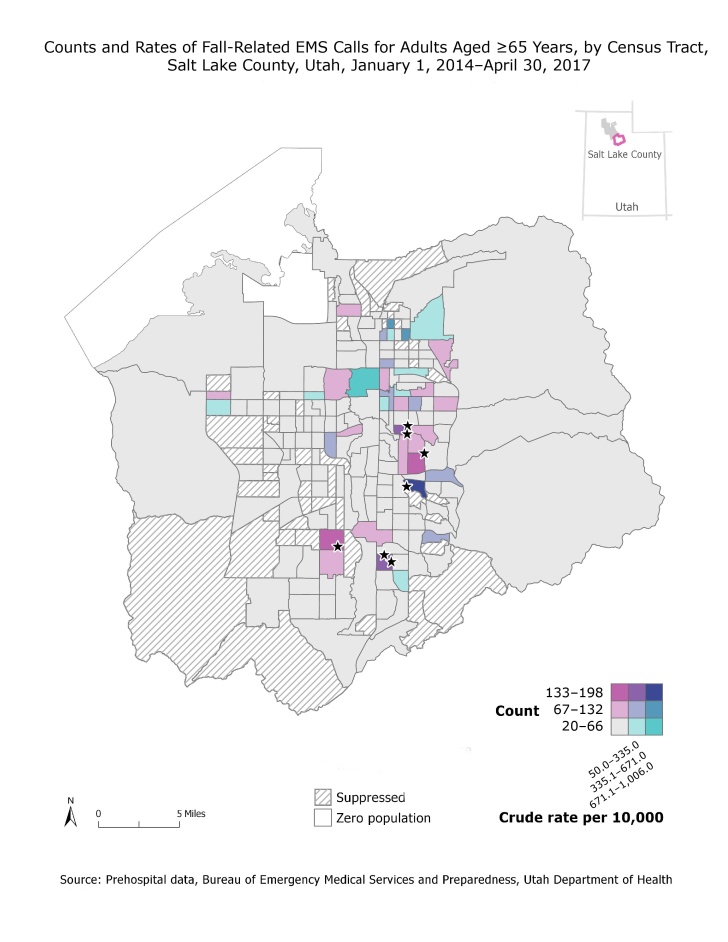
The map displays counts and rates, by census tract, of fall-related EMS calls among adults aged 65 or older in Salt Lake County, Utah, from January 1, 2014, through April 30, 2017. Stars indicate senior living residences with high fall burden. Data were suppressed in areas that had fewer than 20 falls or the relative standard error of the crude rate of fall-related EMS calls was higher than 30%. Abbreviation: EMS, emergency medical services.

## Background

Every year in the United States, more than 25% of people aged 65 or older fall at least once (1), and these injuries are associated with high rates of illness and death (2). Physiological age-related changes such as reduction of sight, hearing, and muscle strength are major causes of falls among older people (3). Among Salt Lake County residents aged 65 or older, in 2014 the fall injury emergency department encounter rate was 458.6 per 10,000 (4) and the fall injury hospitalization rate was 130.0 per 10,000 (5), and in 2016 the fall mortality rate was 5.4 per 10,000 (6).

As part of ongoing fall prevention activities, the Salt Lake County Health Department hosts the evidence-based Stepping On program for seniors. To better target this and other fall prevention programs to reduce rates of fall-related illness and death, we mapped dispatched emergency medical services (EMS) calls for falls and fall-related injuries among adults aged 65 years or older and identified areas with high prevalence.

## Methods

We extracted data on EMS calls from the Utah prehospital reporting system that had an incident address in Salt Lake County; a date of incident from January 1, 2014, through April 30, 2017; and a dispatch report of 1) a fall or 2) an unconscious/fainting or unknown problem/person down. Those with a dispatch report of a fall were assumed to be correctly classified as fall-related. Narratives of those with a dispatch report of an unconscious/fainting or unknown problem/person down were searched by using SAS version 9.4 (SAS Institute, Inc) for terms suggesting evidence of a fall, such as “fall,” “fell,” or “GLF” (ground level fall). Calls were excluded if the narrative included terms indicating no fall, such as “no fall,” “negative fall,” “not sustain a fall,” “denies (any) fall,” or “not suffer a GLF.”

The final data set included 14,824 fall-related injuries. Of those, 93% could be geocoded (96% of those geocoded with match score ≥90) and aggregated to the census tract level by using ArcGIS Pro 2.0 (Esri). Crude incidence rates were calculated by using American Community Survey 5-year population estimates for adults aged 65 years or older from 2014 through 2016, and mapped by census tract. Both counts and rates were classified by using equal intervals. Fall injury points were overlaid on census tract rates, and Google Maps (Google LLC) was used to explore neighborhoods with high counts or crude rates to identify facilities where fall prevention activities may be targeted.

Institutional review board (IRB) approval was obtained for the EMS data set from the Utah Department of Health IRB Committee.

## Findings

Fall injury counts among adults aged 65 years or older were highest in census tracts in southeast and southwest Salt Lake County. Fall injury rates among adults aged 65 years or older were highest in census tracts in north-central and southeast Salt Lake County. Seven facilities were identified as locations of falls in these high-count or high-rate areas; all were mixed-level senior living residences (eg, independent living, assisted living, memory care). One census tract with a high rate did not have a senior living facility; most of these falls occurred in individual homes because of safety hazards. All census tracts with a high count had at least one senior living facility in which most falls occurred.

## Action

Results were used by community partners to secure pilot funding for the Otago Exercise Program, and they are currently being used to target Stepping On and Otago programs, collaborate with Salt Lake County Aging and Adult Services’ Meals on Wheels program to better reach the senior population vulnerable to falls, develop one-on-one prevention programs at sites with a high prevalence of falls, implement collaborative fall prevention programs with EMS community paramedicine programs, and evaluate program interventions. Interventions target individuals and include easily modifiable risk factors such as muscle strengthening and balance retraining exercises, medication review, vision and hearing checks, and improving safety around the home. However, public health would do well to partner with other sectors, such as city planning, to improve the built environment for seniors.

Geospatial data can be challenging to interpret, and various analyses and visualizations should be assessed together for the most accurate picture. Assessing rates without also examining counts may lead to inappropriate resource allocation because of the size of the population aged 65 or older in certain census tracts. For example, one census tract had a high rate but low count because the population aged 65 years or older in this tract was small. Similarly, census tracts with a low rate and high count indicate areas where the population aged 65 or older is large. Resources allocated to high-rate/low-count tracts may have a lesser impact in reducing the burden of falls than resources allocated to low-rate/high-count tracts. Ultimately, program managers found count data most useful for targeting resources to locations. In the future, it would be useful to compare locations by calculating rates by facility.

Limitations of this project include incomplete 2017 prehospital data resulting from a reporting delay, missing 2015–2016 prehospital data from one EMS agency because of data submission issues, and potential misclassification of incidents as fall-related or not fall-related.
